# Role of Robot-Assisted Pelvic Surgery

**DOI:** 10.1100/tsw.2009.54

**Published:** 2009-06-12

**Authors:** Iqbal Singh, Ashok K. Hemal

**Affiliations:** Department of Urology, Wake Forest University School of Medicine, And Wake Forest University Baptist Medical Center, Medical Center Boulevard, Winston-Salem, NC, USA

**Keywords:** robot, pelvic surgery, urinary incontinence in female, robot in urology, cystectomy, genitourinary fistulas, robot-assisted anterior exenteration, robot-assisted ureteral reimplantation, robot-assisted sacrocolpopexy, robot-assisted myomectomy, robot-assisted hysterectomy

## Abstract

The purpose of this study was to assess the current role of robot-assisted urological surgery in the female pelvis. The recently published English literature was reviewed to evaluate this role, with special emphasis on reconstructive procedures. These included colposuspension for genuine female stress urinary incontinence, repair of female genitourinary fistulas, ureterosciatic hernias, sacrocolpopexy for vault prolapse, ureterolysis and omental wrap for retroperitoneal fibrosis, ureteric reimplantation, and bladder surgery. To date, a wide spectrum of urogynecological reconstructive procedures have been performed with the assistance of the surgical robot and have been reported worldwide. Currently, a number of female pelvic ablative and reconstructive procedures are technically feasible with the aid of the surgical robot. While the role of robot-assisted surgery for bladder cancer, ureterolysis, ureteric reimplantation, repair of genitourinary fistulas, colposuspension, and sacrocolpopexy is nearly established among urologists, other procedures, such as myomectomy, simple hysterectomy, trachelectomy, and Wertheim's hysterectomy, are still evolving with gynecologists. The advantages of robot assistance include better hand-eye coordination, three-dimensional magnified stereoscopic vision with depth perception, intuitive movements with increased precision, and filtering of hand tremors. For most of the currently performed procedures in selected patients, the robot-assisted surgical outcomes appear to be relatively superior as compared to an open and purely laparoscopic surgical procedure.

## INTRODUCTION

Traditional, big-incision open surgery has given way to minimally invasive surgery and laparoscopy in many areas of urological surgery. Laparoscopic surgery entails making small and precise movements within a confined space, such as the bony pelvis, making it not only technically demanding, but also time consuming as both of these contribute to a surgeon's fatigue. In addition, the surgeon's tremor tends to amplify with the use of the long laparoscopic instruments. The flat-screen monitor used in laparoscopic surgery displays only a two-dimensional image, which lacks depth perception. The range of movements with laparoscopic urological instruments and surgery are limited in most situations due to their basic design and space constraints. Advanced laparoscopic urological procedures have evolved slowly and are currently limited to a few technically advanced centers. Advanced laparoscopy also requires the acquisition of special surgical skills and traditionally requires a long training curve. The availability of a "robot interface" has helped many urologists overcome these problems, and has also led to an improved transfer of laparoscopic surgical skills among both postgraduate residents of urology as well as practicing urologists[1].

The Robot Institute of America defines a robot as "a programmable, multifunctional manipulator designed to move materials, parts, tools, or specialized devices through variable programmed motions for the performance of a variety of tasks." Robots designed for surgical application possess three main areas of supremacy over humans, namely: (1) spatial precision and accuracy, (2) reliability, and (3) consistency coupled with repeatability[2]. The "robot" is a computer system–controlled mechanical interface with a multitude of sensors and motors that transmit faster and repetitive movements with greater precision without the risk of fatigue in all three situations. The "robot" is endowed with an amphidextrous joint (EndoWrist®, 7 degrees of freedom), which reduces the error rate required to complete a particular designated task[3].

The da Vinci® and Zeus™ surgical robot systems are two such examples of the online master-slave systems in which the surgeon is placed at a remote console and the operative site is displayed on a highly magnified (10–15 times), three-dimensional realistic display (stereoscopic depth perception). The surgeon's movements are transmitted via a "data glove" to the robot arms via computerized translation interphase. Robot-assisted laparoscopic surgical technology is well suited to facilitating and enabling major advanced reconstructive, as well as ablative, urological and urogynecological procedures within the limited confines of the pelvis. This manuscript describes the recent advances in the field of robot-assisted surgery and its impact on urological surgery, especially in the area of female pelvic and female urologic surgery. Robot-assisted laparoscopic ablative pelvic procedures that have been performed to date include radical cystectomy with or without intracorporeal urinary diversions (ileal conduit, ileal orthotopic neobladder, or the rectosigmoid-Mainz-II pouch), anterior exenteration, uterine myomectomy, and hysterectomy. Robot-assisted pelvic reconstructive surgical procedures performed include repair of vesicovaginal fistulas (VVF), vesicouterine fistulas (VUF), ureterovaginal fistulas (UVF), urethrocolposuspension, pelvic prolapse, ureterolysis, and ureteric reimplantation.

## Robot-Assisted Repair of Urinary Fistulas (VVF, UVF along with Ureteroneocystostomy, and VUF with or without Hysterectomy)

The first report of robot-assisted laparoscopic repair of VVF was initially described by Melamud et al. in 2005[4]. Later, Sundaram et al. published a report describing their technique of a purely robot-assisted repair of VVF in five of their cases[5]. These workers stressed the importance of accurate and ergonomic placement of ports as one of the factors vital to the success of any robot-assisted laparoscopic surgery, so as to avoid instrument collision, cluttering, and technical mishaps[6]. Elsewhere, Hemal et al. also separately described their robot-assisted laparoscopic technique for repair of recurrent supratrigonal VVF[7].

### *Technique*

Routine bowel preparation and prophylactic antibiotics are initiated a day before the surgery. Under general anesthesia, nasogastric intubation and bilateral ureteric catheterization is performed with urethral catheterization, and the patient is tilted in a low lithotomy position. Initial vaginoscopy and cystoscopy are performed to ascertain the location and size of the fistula and its relationship with the ureteral orifices. The pneumoperitoneum is established in the standard laparoscopic manner and a five-port technique is used as described earlier[5]. Gas leakage is minimized by placing the Foley catheter across the vaginal fistula into the bladder, placing it on sustained gentle traction, and by snugly packing the vagina with moist gauze. These authors typically use the following instruments essentially for this procedure: monopolar scissors, EndoWrist-PK dissector, and a large needle driver. The robot is docked, and initial peritoneoscopy (for surgical orientation) and adhesiolysis is performed in cases wherein abdominal adhesions are present. Robot-assisted VVF dissection is initiated. After identifying the bladder, a posterior cystotomy is created at about 3 cm above the VVF. This is then advanced to encircle the VVF. This effectively demonstrates the bladder and the fibrous tissue rim around the fistula. This fibrous tissue is then excised and the ends are freshened; this effectively separates the bladder from the vagina. The ureteric orifices are identified with the indwelling ureteric catheters, which are removed if not needed. Adequate dissection and freshening of the fistulous margins are performed so as to allow tension-free suturing. A double-layered 3-0 vicryl interrupted suturing for closure of the vagina (horizontal closure) and bladder (vertical closure) is performed with omental interposition, with the aid of the robot. Bladder closure is confirmed by gently distending it with 200 ml of dilute povidone iodine solution. The right iliac port site is used to insert a temporary abdominal drain (3 days). An indwelling Foley catheter is kept for 10 days. Patients can generally be discharged on day 3 or 4. A voiding study is usually performed on day 10.

In the robot-assisted repair of VVF described by Hemal et al., these authors concluded that even recurrent supratrigonal fistulas could be repaired successfully[7]. Laparoscopic repair of genitourinary fistulas is a technically demanding, complex, and skilled procedure, and for this reason, it has not gained widespread support among urologists and gynecologists. Loss of pneumoperitoneum during the robot-assisted reconstruction and repair of VVF is an area of concern; this can, however, be minimized by using another device (an end-to-end anastomotic sizer with an occlusion balloon) as described by Sears et al. in 2007[8]. The da Vinci robot-assisted surgical interphase facilitates precise identification of tissue planes, accurate dissection, and trouble-free suturing deep inside the pelvis, with a magnified three-dimensional perspective, with the surgeon sitting comfortably at a console. A major advantage of using the robot is the ease of performing precise anatomical dissection in greater depths due to the EndoWrist feature of the instruments in facilitating an otherwise demanding and complex laparoscopic task[9]. Sears et al. concluded that using the da Vinci robot interface substantially facilitated the double-layered closure of the bladder, with interposition of the omental graft and closure of the vagina in their patients undergoing a repair of VVF[8]. A robot-assisted laparoscopic approach to repair of VVF without intentional cystotomy has also been described successfully elsewhere by other workers[10]. Other complex genitourinary fistulas like UVF (along with ureteroneocystostomy)[11] and VUF (with or without hysterectomy)[12] have also been successfully described and repaired with the aid of robotic assistance.

## Robot-Assisted Bladder Neck Colposuspension

Bladder neck suspension is principally indicated for correction of urodynamically proven, genuine stress urinary incontinence where conservative measures have failed to provide symptomatic relief. Laparoscopic colposuspension is the preferred minimally invasive current procedure for the correction of urinary incontinence associated with a low urethral closing pressure, low valsalva leak point pressure, and a hypermobile urethra[13]. Prior operative procedures on the bladder neck and urethra may not necessarily contraindicate surgery unless the urethra is rigid and adherent. Currently, solo surgery may be technically feasible with the aid of robot assistance, without the need for an additional assistant surgeon[14].

### *Technique*

Robot-assisted colposuspension is performed using the technique as previously outlined elsewhere by Tanagho[15]. An extraperitoneal three- or four-port technique is used[16] or, alternatively, an intraperitoneal technique can also be used. In the extraperitoneal robot-assisted approach, the patient is placed in a 45° Trendelenburg tilt. The extraperitoneal space is developed by balloon dissection through a small infraumbilical incision and a three-dimensional video camera is inserted via this robot port. Two additional lateral (pararectal) robot ports are introduced under vision at about 5 cm, inferolateral to the umbilicus at the level of both the anterior superior spines. A fourth 10-mm port (assistant) is inserted 5 cm superolateral to the right anterior superior iliac spine. The patient is now placed in a Trendelenburg tilt and the robot is docked. The robot bipolar forceps and hook are used to perform the initial extraperitoneal dissection in the space of Retzius to delineate the lateral bladder wall, Cooper's ligament, and paravaginal tissues, taking care to stay about 3–4 cm above the pubis so as to avoid any inadvertent entry into the bladder. The Cooper's ligament is identified and cleared along the superior aspect of the pelvic bone. Care is also taken to stay about 2 cm lateral to the urethra, with simultaneous lateral paraurethral elevation being maintained with the aid of the assistant's fingers inserted into the vagina.

A large EndoWrist robot needle driver is used to place at least three sutures through the paraurethral or paravaginal tissues lateral to the midurethra and through the Cooper's ligament on each side. The slipknot is then tied extracorporeally. An assistant's vaginal hand or a Hegar dilator is used to elevate the ipsilateral vaginal fornix and check for any excessive tension on the sutures. A retroperitoneal drain is placed at completion of the procedure and the robot is undocked. A cystoscopy is routinely performed in order to exclude inadvertent bladder injury following placement of the sutures.

A robot-assisted laparoscopic colposuspension facilitates accurate suturing of the paravaginal tissues and the Cooper's ligament. It also allows the procedure to be completed comfortably within 2–2.5 h with negligible blood loss (usually less than 20 ml). Robot-assisted laparoscopic colposuspension facilitates a shorter hospital stay (2–3 days), while providing effective continence rates. The relative safety, feasibility, and efficacy of robot-assisted laparoscopic colposuspension has been successfully demonstrated by Khan et al.[16]. The difficulties associated with laparoscopic colposuspension, such as restricted instrument maneuverability in the pelvis, limited two or three degrees of freedom, and complexity of placing the sutures accurately through the iliopectineal ligament, are simplified and overcome by use of the robot interface. In the opinion of these authors, robot-assisted colposuspension may be currently indicated as the minimally invasive alternative surgical procedure of choice, as opposed to open and/or laparoscopic colposuspension in conjunction with other gynecologic procedures, such as hysterectomy or repair of vault prolapse, for the management of patients with pure SUI. Alternatively, similar cases of SUI can also be managed by other simpler options, e.g., midurethral slings.

## Robot-Assisted Laparoscopic Ureterolysis

Idiopathic retroperitoneal fibrosis (IRPF) has been successfully managed in the past by other surgeons by laparoscopic ureterolysis[17,18,19]. Kavoussi et al. reported their first case of laparoscopic ureterolysis in 1992[19]. Recently, Mufarrij et al.[20] published and described their maiden technique of robot-assisted ureterolysis. Subsequently, Stifelman et al.[21], along with other surgeons[22], also successfully reported and described their technique of robot-assisted ureterolysis, retroperitoneal biopsy, and laparoscopic omental wrapping of the ureter for the management of ureteral obstruction due to IRPF[23,24]. These surgeons also successfully described and reported the use of robot-assisted laparoscopic surgery for performing ureterolysis along with ligation of the ovarian vein for the surgical management of the ovarian vein syndrome (intermittent ureteric obstruction due to the thrombosed left ovarian vein)[22].

### *Technique*

After a preliminary cystoscopy, retrograde pyelography, and stenting, the patient is placed in a left lateral position (for right ureterolysis) and vice versa. A five-port technique, as separately described elsewhere by Mufarrij et al.[20], is used. A 12-mm trocar and two robot 8-mm trocars are placed in a "V"-shaped manner, along with two additional assistant trocars (12 and 5 mm) placed in the midline above the camera port. Stifelman et al.[21] described their five-port robot-assisted laparoscopic technique in which they placed their initial 12-mm port lateral to the umbilicus along with two 8-mm robot ports placed in the midclavicular line above and below the first port. Two additional 5- and 12-mm ports were also placed across the midline for the assistant surgeon on the side opposite to the robot trocars.

The robot is docked and initial peritoneoscopy is performed. The white line of "Toldt" is incised in the usual manner so as to completely mobilize the right or left colon, exposing the ipsilateral ureter and retroperitoneum. After mobilizing the ureter proximal and distal to the area of maximal periureteral fibrosis (encasement) and obstruction, curved Pott's scissors (intuitive surgical) are used to perform an anterior ureterotomy deep down to its adventitia. Subsequently, the entire ureter is freed circumferentially using the Maryland bipolar forceps (fenestrated), Pott's scissors, and by applying retraction on the vessel loops along with suction by the assistant surgeon. The fibrotic mass is excised and sent for biopsy. The robot is undocked and laparoscopic omental mobilization is performed to enable omental wrapping of the lateralized diseased ureteric segment in the standard manner. The procedure is terminated by inserting a retroperitoneal flank drain and Foley urethral catheter. These are removed after 48 h and patients are usually discharged by the third postoperative day.

The operating time, blood loss, and hospital stay for robot-assisted ureterolysis is comparable with bilateral laparoscopic ureterolysis (less than 4 h, 10 ml, and 3 days, respectively)[18,20]. Follow-up renal scans have demonstrated relief of obstruction in up to 92% of such cases, with the overall reported success rates approaching 87%[20,22]. Robot-assisted ureterolysis has distinct advantages over the laparoscopic technique[20,21]. In the opinion of these authors, robot assistance provides an unparalleled clarity and ease of dissection (using the Pott's scissors) that enables a complete freedom of mobility in six planes that is not possible with traditional laparoscopy. The second advantage of using the da Vinci surgical robot system (DSRS) is the incorporation of superior three-dimensional stereoscopic vision and tremor-filtered movements that provide a relatively secure surgical procedure and a stable operative field.

## Robot-Assisted Ureteric Reimplantation

Laparoscopic extravesical[25] and transvesical[26] ureteral reimplantation for vesicoureteral reflux using the Lisch Gregor's principle of reimplantation has been described earlier by other workers[27]. With the entry of the DSRS into the domain of laparoscopic urology, the feasibility of performing ureteral reimplantation and approaching the lower ureter using a robot-assisted laparoscopic approach has also been described by others[28,29]. One of the largest published series of robot-assisted laparoscopic ureteral reimplantation that has been reported to date was described by Patil et al.[30].

### *Technique*

Casale et al.[28] recently described their extravesical technique of robot-assisted laparoscopic ureteral reimplantation for vesicoureteral reflux in some of their pediatric patients. After the initial cystoscopy and ureteral catheterization, a transperitoneal three-port technique is used. The umbilical trocar is used to insert the robot camera via the umbilical port, the robot is docked, and after the initial peritoneoscopy, two additional ports are inserted on the lateral border of the rectus at the level of anterior superior iliac spine. The peritoneal bladder reflection is incised to expose the posterior bladder wall and the distal ureter/s is/are mobilized above and below the level of uterine vessels. The pelvic plexus is carefully identified, while mobilizing the ureter at the hiatus and retracting it medially. Circumferential ureteral hiatus dissection is performed with the pelvic nerves under vision. In 2008, Patil et al.[30] also reported a series of robot-assisted ureteral reimplantations using the psoas hitch procedure in a multi-institutional and multinational evaluation. After catheterization with the patient in dorsal lithotomy position, they created a pneumoperitoneum and placed four trocars. The 12-mm camera port was placed 5 cm supraumbilically in the midline, followed by two 8-mm ports placed pararectally 2 cm below the level of the first port. The fourth accessory 5-mm port was placed on the contralateral ureter side just above the iliac crest. The DSRS was docked after a further 20° Trendelenburg tilt. Next, the ipsilateral hemicolon and the diseased ureter were dissected and mobilized, preserving the blood supply. After placing an anchoring ureteric suture, the diseased ureter was then transected just above the strictured segment. The bladder was distended with saline to facilitate dissection of the bladder dome; the peritoneum was also incised to approach the superior aspect of the psoas muscle. The bladder was then hitched to the psoas muscle with a large needle driver by placing two sutures about 2 cm apart. A 7-cm long cystotomy of the bladder dome was carried out, keeping the bladder wall open by applying traction on the two abdominal wall sutures. A submucosal tunnel was created by meticulous dissection (using the robot scissors) and the mobilized ureter was pulled through it by placing traction on the ureteric anchoring suture. The ureter was then spatulated, anchored to the detrusor muscle, and the ureteroneocystostomy was performed over a retrograde JJ stent. The procedure was terminated after closing the bladder in two layers.

The overall mean operating time was about 2.5 h (inclusive of robot console time of 2 h), with an estimated blood loss of 50 ml and a hospital stay of 4 days[30]. This has been shown to be certainly lower as compared to the open as well as purely laparoscopically operated cases[31]. The initial data and the short-term results of robot-assisted laparoscopic ureteroneocystostomy appear to be excellent in terms of the overall operating time, mean analgesic requirement, average hospital stay, and estimated blood loss[30,31]. However, the long-term data are yet to emerge. Laparoscopic ureteral reimplantation remains a technically complex procedure, demanding a high degree of proficiency and surgical expertise. This is primarily due to the intricate nature of performing an intracorporeal, laparoscopic, nonrefluxing, tunneled ureteroneocystostomy. Robot assistance provides an unparalleled maneuverability, accurate submucosal dissection, and facilitates tunneling of the ureter and its subsequent suturing; all these are highly complex laparoscopic tasks. However, the chief advantage of use of the robot appears to be the relative ease of suturing the ureteroneocystostomy, which is otherwise a highly skilled, purely laparoscopic procedure associated with a lengthy learning curve for most urologists. The technique of robot-assisted laparoscopic ureteral reimplantation is currently still continuing to evolve.

## Robot-Assisted Urogynecological Procedures

A wide variety of robot-assisted laparoscopic gynecologic surgeries, such as myomectomy, total and supracervical hysterectomy, trachelectomy, ovarian cystectomy, colposacropexy, and Moskowitz procedure, are technically feasible and have been successfully described by many authors and coworkers[32,33,34,35,36,37].

## Robot-Assisted Sacrocolpopexy and Sacrouteropexy

Sacrocolpopexy is used to correct vaginal vault prolapse in the absence of the uterus, while sacrouteropexy is used to correct advanced pelvic organ prolapse in the presence of the uterus. These surgical procedures are generally combined along with a urinary anti-incontinence procedure like Burch colposuspension. Traditionally, these procedures have been performed as open abdominal procedures, but over the last several years, minimally invasive laparoscopic sacrocolpopexy has emerged and evolved successfully as a safe and durable procedure for the treatment of female pelvic organ prolapse[32]. Recently, some workers also reported the feasibility, safety, as well as early advantages of performing robot-assisted laparoscopic sacrocolpopexy and/or sacrouteropexy[33,34,35,36,37].

## Technique

DiMarco et al. successfully described and reported their technique of da Vinci robot-assisted sacrocolpopexy by using a five-port technique with a transperitoneal approach[33]. The procedure is initiated with the patient in the dorsal lithotomy position. A pneumoperitoneum is established, a 12-mm robot umbilical port is inserted, and two laparoscopic (10 and 5 mm) ports (for retraction) are placed under vision, one through the right subcostal lateral rectus border and the other 10 cm inferolateral to the second port. Two 8-mm robot ports are placed through the inferior rectus border just above the iliac crest. The sigmoid colon is retracted by placing a transabdominal reverse prolene suture for facilitating extracorporeal traction. The patient is tilted into a deep Trendelenburg position; initial peritoneoscopy is performed, followed by laparoscopic vesicovaginal dissection to separate the bladder from the anterior vaginal wall. This is facilitated by the assistant's finger or a retractor in the vagina. Next, the posterior peritoneal fold is incised to mobilize the posterior vaginal wall distally up to the introitus and the peritoneum over the sacral promontory is incised, carefully exposing the shiny sacral periosteum so as not to keep the presacral veins out of harms way. A Y-shaped prolene mesh is inserted via the10-mm port and the robot is docked into position. The robot needle driver is used to transfix the Y part of the mesh first to the posterior and then to the anterior vaginal wall, and the tail of the Y mesh to the sacral promontory. The procedure is terminated by performing laparoscopic culdoplasty (plication of the uterosacral ligament) and closing the posterior peritoneal incision. This retroperitonealizes the posterior mesh. Concomitant vaginal slings are subsequently placed where indicated through a vaginal approach at the midurethra.

Robot-assisted laparoscopic sacrocolpopexy can be successfully accomplished in about 3.5 h without any sequel in a majority of adult women with pelvic organ prolapse[34,35]. In a series of 30 cases of robot-assisted laparoscopic sacrocolpopexy with a 1-year follow-up period, Eliot and Chow reported an overall high level of patient satisfaction without any major complications or recurrences[35]. The procedure could be successfully accomplished in a minimally invasive and least morbid manner akin to open surgery, with the advantage of significantly decreased morbidity and hospital stay. The procedure is facilitated with the aid of robot assistance that helps the surgeon to suture the prolene mesh graft accurately. Robot-assisted laparoscopic sacrocolpopexy is currently viewed as a technically feasible, minimally invasive, durable procedure and a highly effective alternative to a purely laparoscopic sacrocolpopexy. It is being increasingly considered for the surgical management of symptomatic adult women with advanced pelvic organ prolapse.

## Robot-Assisted Myomectomy and Hysterectomy

Robot-assisted laparoscopic myomectomy and hysterectomy have been described as technically safe, viable, and alternative procedures to purely laparoscopic procedures[38,39,40,41]. Mao et al. published a report of robot-assisted laparoscopic myomectomy (preserving the uterus), successfully completing the procedure in 3 h and with a blood loss of about 150 ml[38]. However, currently their numbers are too small to draw pertinent conclusions[39,40,41]. Robot-assisted laparoscopic hysterectomy has been described as a technically feasible procedure that can overcome the potential limitations of performing laparoscopic hysterectomy for benign disease in select cases (such as those with altered pelvic surgical anatomy due to pelvic inflammatory disease/endometriosis leading to a scarred or obliterated cul-de-sac). Advincula and Reynolds[39] successfully reported their results of robot-assisted laparoscopic hysterectomy in six cases, completing the entire procedure in a mean time of less than 4.5 h and with a blood loss under 100 ml. Robot-assisted laparoscopic radical transvaginal trachelectomy (excision of cervix uteri) has also been successfully described as a feasible procedure in patients with a past history of hysterectomy[37,40,41]. Bartos et al.[41] also described the safety and technical feasibility of performing a wide range of gynecological ablative oncological procedures, such as total robot-assisted laparoscopic hysterectomy, robot-assisted vaginal hysterectomy, and robot-assisted radical vaginal trachelectomy with pelvic lymphadenectomy for uterocervical malignant pathology using the three-arm DSRS. Their operative time was, however, significantly longer (22 vs.12 h) vis-à-vis laparoscopically operated cases[41].

Robot-assisted laparoscopic gynecological surgery helps to overcome many of the limitations towards performing a purely laparoscopic approach in both benign and malignant pathologies of the cervix and uterus. Robot assistance has also been successfully used for managing early cervical cancer (radical parametrectomy) and fertility-preserving trachelectomy has been shown by some workers to be technically feasible, secure, and easier to perform as compared to the purely laparoscopic approach[42]. This has also been found to be true even for advanced and recurrent cervical cancer where complex procedures, such as robot-assisted laparoscopic retroperitoneal lymphadenectomy and robot-assisted laparoscopic radical pelvic exenteration, have been found to be technically easier to carry out with the aid of a surgical robot[42]. Although robot assistance for laparoscopic treatment of gynecological benign and malignant diseases is safe and feasible in terms of outcome goals, cost and prolonged operative times currently remain the major limiting factors. Currently, the routine use of robot assistance in performing gynecological ablative oncological procedures may not be justified. However, a possible role for the use of robot assistance in select (complicated) cases of myomectomy and trachelectomy may be advantageous vis-à-vis a purely laparoscopic procedure. Robot-assisted surgery may also provide the much-needed link to overcome the gap between conventional laparotomy and laparoscopy in the female pelvis for several gynecological disorders requiring hysterectomy.

## CONCLUSIONS

The outstanding advantages of robot-assisted minimally invasive surgery over conventional surgery are yet to emerge with its full global potential due to current cost constraints. Table 1[4,5,6,7,14,15,16,17, 18,19,32,33,34,35,36,37,38,39,43,44,45,46,47,48,49] shows a list of a current spectrum of cases that have been performed with the aid of robot-assisted laparoscopic surgery in the female pelvis. In the future, robot-assisted surgeries may radically alter forever the way surgery is performed. Thus, it is vital for modern-day urologists to keep abreast of the new emerging technologies and their limitations, and to explore the possibility of incorporating them into day-to-day surgery[45]. Currently, the entire gamut of pelvic organ prolapse procedures (anterior prolapse, posterior prolapse, apical prolapse, culdoplasty, enterocele excision closure, uterosacral ligament suspension, and sacrocolpopexy) can be accomplished with the aid of the robot[46]. Recently, even some vaginal reconstructive procedures, such as robot-assisted sigmoid vaginoplasty, have also been shown to be technically feasible[49].

Robot-assisted sacrocolpopexy has proven to be a viable option compared to open or transvaginal surgery, with a significant decline in postoperative morbidity[47]. Robot-assisted laparoscopic surgery is rapidly emerging as a minimally invasive alternative to pure laparoscopy in gynecologic oncology for the surgical management of uterine and cervical cancer, and currently its role in ovarian cancer is being explored[48].

At present, there is an explosive growth in the number of ongoing studies that evaluate the feasibility and outcome of using the DSRS in several pelvic urological and urogynecological procedures[48]. Robot-assisted reconstruction of the lower urinary tract is still in its infancy, but is set to further expand and evolve. Robot-assisted psoas hitch, boari flap, and ileal ureter, along with distal ureterectomy, have also been successfully performed and reported[30]. There are several reports currently documenting the successful outcome of complex urological ablative and reconstructive procedures in the female pelvis performed with the assistance of the robot in humans, attesting to their safety, efficacy, and feasibility. Overall, the DSRS has been shown to be extremely beneficial to urologists undergoing a transition from open to laparoscopic or minimally invasive surgery. In a study, it was demonstrated that even laparoscopically naïve (although experienced) open surgeons can successfully transfer their open surgical skills to a laparoscopic environment with a limited number of cases by using a robot assistance[50]. This is expected to create a surge in the global demand for acquiring surgical robots in the near future, with the

**TABLE 1**
**List of Laparoscopic Procedures Performed in the Female Pelvis With and Without Robotic Assistance**

| Organ | Ablative | Reconstructive |
|---|---|---|
| Bladder | Diverticulectomy; cystectomy — partial, simple, radical[43,44] | Orthotopic neobladder, bladder neck suspension[14,16], VVF[4,5,7], and VUF repair |
| Ureter | Distal ureterectomy | Ureterolysis[17,18,19] ± omental wrapping (for IRPF)[20,24], ureteroneocystostomy[28] ± psoas hitch[30] ± Boari flap, UVF[11], and ureterouterine fistula[12] repair |
| Vagina | Anterior pelvic exenteration[45] | Colposuspension[16], sacrocolpopexy[32,33,34,35], vaginal cuff suturing, enterocele repair[46], sigmoid vaginoplasty |
| Uterus, cervix, fallopian tube | Myomectomy[38]; endometriosis resection; hysterectomy — total, radical[48], supracervical; parameterectomy[42]; +trachelectomy[40] | Sacrouretropexy[36] , tubal reanastomosis[37,39] |
| Ovary | Ovarian cystectomy | — |
| Lymph nodes | Radical pelvic lymphadenectomy[45,49] | — |

aim of performing technically difficult reconstructive laparoscopic urological procedures in the least minimally invasive manner both within and beyond the confines of the pelvis.
